# CAR-T in Cancer Treatment: Develop in Self-Optimization, Win-Win in Cooperation

**DOI:** 10.3390/cancers13081955

**Published:** 2021-04-19

**Authors:** Feifei Guo, Jiuwei Cui

**Affiliations:** Cancer Center, The First Hospital of Jilin University, 71 Xinmin Street, Changchun 130021, China; guoff19@mails.jlu.edu.cn

**Keywords:** chimeric antigen receptor T cell therapy, cancer immunotherapy, CAR structure, combined therapy

## Abstract

**Simple Summary:**

Chimeric antigen receptor (CAR)-T cell therapy has exhibited good application prospects in the treatment of hematologic malignancies. However, there are still many unsolved problems, such as the limited antitumor effect of CAR-T on solid tumors and the potential risk of CAR-T therapy in clinical applications. In order to meet these challenges, more and more solutions are proposed. Therefore, in this review, we have discussed the recent breakthroughs in CAR-T therapy for cancer treatment, with an emphasis on the potentially effective CAR-T modifications and combined strategies.

**Abstract:**

Despite remarkable achievements in the treatment of hematologic malignancies, chimeric antigen receptor (CAR)-T cell therapy still faces many obstacles. The limited antitumor activity and persistence of infused CAR-T cells, especially in solid tumors, are the main limiting factors for CAR-T therapy. Moreover, clinical security and accessibility are important unmet needs for the application of CAR-T therapy. In view of these challenges, many potentially effective solutions have been proposed and confirmed. Both the independent and combined strategies of CAR-T therapy have exhibited good application prospects. Thus, in this review, we have discussed the cutting-edge breakthroughs in CAR-T therapy for cancer treatment, with the aim of providing a reference for addressing the current challenges.

## 1. Introduction

**Figure 1:**
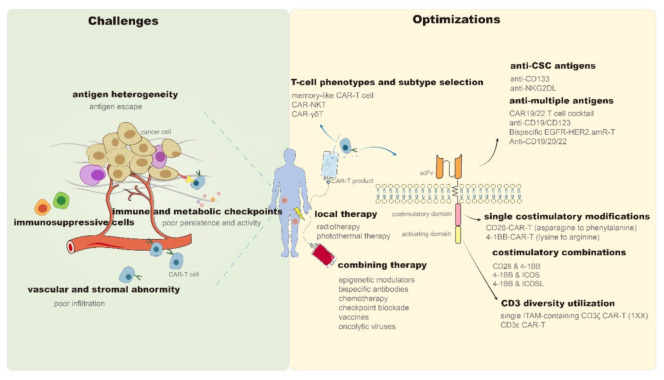
Challenges and optimizations of CAR-T persistence and antitumor properties. CAR—Chimeric antigen receptor; NKT—Natural killer T; γδT—gamma delta T; CSC—Cancer stem cells; CD—Cluster of differentiation; NKG2DL—Natural killer group 2 member D ligand; EGFR—Epidermal growth factor receptor; HER2—Human epidermal growth factor receptor 2; amR—Antibody mimic receptor; scFv—Single-chain variable fragment; 4-1BB (TNFRSF9)—Tumor necrosis factor receptor superfamily 9; ICOS—Combining inducible costimulatory; ICOSL—Combining inducible costimulatory ligand; ITAM—Immunoreceptor tyrosine-based activation motif.

In 2017, chimeric antigen receptor (CAR)-T cell products, represented by Kymriah (CTL019) [1] and Yescarta (KTE-C19) [2], were approved for marketing by the Food and Drug Administration (FDA), which highlights the therapeutic potential of CAR-T in hematologic malignancies. However, despite being a promising antitumor candidate, CAR-T still faces many problems as it continues to encounter sequent obstacles in the treatment of solid tumors. It is difficult for CAR-T cells to properly migrate and infiltrate into solid tumor sites due to the chemokine mismatches between CAR-T and tumor cells [3] and the presence of abnormal vascular [4] and stromal [5] components in the tumor microenvironment (TME). Subsequently, CAR-T cells entering tumor tissues compete with diverse immunosuppressive cells (e.g., regulatory T cells and myeloid-derived suppressor cells) [6,7], immune checkpoints (e.g., programmed death-ligand 1 [PD-L1]) [8], and metabolic checkpoints (e.g., hypoxia and glycolysis) [9,10] in the TME. Even after successfully entering tumor tissues, these CAR-T cells have to cope with problems such as tumor antigen heterogeneity and antigen escape [11,12]. Moreover, activity reduction and persistence limitation of infused CAR-T products may be the leading cause of tumor recurrence in hematologic and solid tumors [13]. The inevitable immune-mediated toxicity, manifested as cytokine release syndrome (CRS) and immune effector cell-associated neurotoxicity syndrome (ICANS), is also a critical problem for the clinical application of CAR-T [14]. Although mild patients may present only self-limited symptoms such as fever and hypotension, life-threatening organ damage and death can also occur, similar to that reported in the P-PSMA-101 study or with the UCARTCS1 product [15]. Although CAR-T products of good manufacturing practice (GMP) grade are currently available, the CAR-T manufacturing process still needs to be improved to increase clinical access [16]. Methods of obtaining T cell isolation, activation, expansion, and genetic modification more effectively and economically are worth exploring [17,18]. The success of CAR-T therapy in cancer treatment traps it into obstacles. Recently, more effective solutions have been developed and reported. Therefore, in this review, we have discussed and summarized the recent remarkable improvements and breakthroughs in CAR-T therapy to address the existing challenges (Figure 1).

## 2. Enhanced Persistence and Antitumor Properties of CAR-T Therapy

### 2.1. Optimization of CAR-T Structures

#### 2.1.1. Optimization of the Extracellular Domain

The single-chain variable fragment (scFv), designed to identify tumor antigens, is the major component of the CAR-T extracellular segment. Antigen heterogeneity is an important factor hindering CAR-T efficacy in cancer treatment. The varying antigen expression densities and the simultaneous existence of different antigens have been reported to be critical for tumor escape or recurrence [11]. Therefore, promoting CAR-T-specific antigen recognition may be helpful for augmenting CAR-T efficacy in cancer treatment.

##### Optimization of the Extracellular Domain

A fraction of cells with stem-like properties in solid tumors called cancer stem cells (CSCs) are widely known for their association with tumor-heterogeneity evolution and resistance to conventional anti-tumor therapies [19]. Given this, a growing number of CSC-associated antigens have been selected to enhance CAR-T function. Cluster of differentiation (CD)133 is a common marker of CSCs in multiple aggressive solid tumors, and Vora et al. constructed a CAR-T-targeting CD133 (CART133) and confirmed its superior efficacy in patient-derived glioblastoma (GBM) xenograft models, further indicating that CART133 in the treatment dose not induce toxicity in normal CD133^+^ hematopoietic stem cells and progenitor cells in humanized CD34^+^ mouse models [20]. Moreover, the overexpression of natural killer group 2 member D ligands (NKG2DLs), such as the major histocompatibility complex class I chain-related gene A/B (MICA/B) and UL16 binding protein (ULBP), has been reported in GBM stem cells (GSCs), and CAR-T expressing natural killer group 2 member D (NKG2D) has been shown to exhibit effective tumor-elimination effect on GBM models, with insignificant treatment-related toxicity [21].

##### Targeting Simultaneously on Multiple Antigens

Targeting two or more tumor antigens is an important strategy. Sequential administration of different CAR-T cells targeting individual tumor antigens was first investigated and showed good results in clinical studies [22]. One pilot study (ChiCTR, number ChiCTR-OPN-16008526) conducted in 89 refractory/relapsed (r/r) B-cell malignancy patients (51 acute lymphoblastic leukemia patients and 38 non-Hodgkin lymphoma patients) showed that the sequential infusion of anti-CD19 and anti-CD22 CAR-T cells (CAR19/22 T cell cocktail) exhibited a remarkable anti-tumor effect (median progression-free survival: 13.6 months and 9.9 months, respectively) and safety (high-grade CRS and neurotoxicity: 22.4% and 1.12% patients, respectively). In addition, only one patient experienced antigen-loss recurrence during follow-up, indicating that the CAR-T cocktail therapy may effectively reduce the recurrence caused by tumor antigen escape [23]. A similar result was observed in 20 children with r/r B acute lymphoblastic leukemia (B-ALL). CAR19/22 T cell cocktail therapy was confirmed to be effective and safe, and it was shown to improve the durability of remission in the long term (ChiCTR, number ChiCTR-OIB-17013670) [24].

The construction of a multi-target CAR on the same T cell is also an effective approach. Dannenfelser et al. screened over 2.5 million dual antigens and approximately 60 million triple antigens across 33 tumor types and 34 normal tissues and predicted that the combinatorial antigens in 2- and 3-antigen AND or NOT logic gates would outperform the current clinical choices to improve tumor discrimination in CAR-T therapies [25]. The corresponding pre-clinical studies of 2- and 3-target CAR-T have been performed in succession with favorable results. Ruella et al. designed a CAR-T with CD19 and CD123 dual expression, and further confirmed its superior activity in B-ALL models compared with single-expressing or pooled combination CAR-T [26]. Furthermore, a novel CAR-T expressing antibody mimic receptor (amR) targeting two different antigens was devised to avoid the potential limitation of the compromised protein folding caused by linearly assembled single chains and the large size of each scFv. For example, bispecific epidermal growth factor receptor (EGFR)-human epidermal growth factor receptor 2 (HER2) amR-T has exhibited significant anti-tumor efficacy in mouse models [27]. Anti-CD19/20/22 CAR-T is an example of a triple-target modification. Fousek et al. created this CAR structure on a single T cell through a tricistronic transgene and found that in addition to CD19^+^ B-ALL models, it can also effectively kill CD19^-^ blasts from recurrent patients after CD19 CAR-T therapy or CD19 knockout primary B-ALL models both in vitro and in vivo [28].

#### 2.1.2. Optimization of the Intracellular Domain

##### Improving Intracellular Activating Domain

CAR structures have been continuously improved from the first generation (1G) CARs consisting only of the CD3ζ signaling domain to 2G and 3G CARs incorporating one or more co-stimulatory domains. Today, 4G and 5G CAR structures with more refined regulatory capabilities have been devised to address the persistence issue and enhance CAR-T efficacy [29].

CD3, comprised of six peptides (CD3γε-CD3δε-CD3ζζ), is a widespread leukocyte differentiation antigen on the T cell surface. The αβ T cell receptor (TCR)-CD3 complex is a well-known critical determinant of antigen recognition and signal transduction of T cells [30]. CD3 diversity has been regarded as an important regulator of CAR-T efficacy. Among these, the CD3ζ cytoplasmic domain containing three immunoreceptor tyrosine-based activation motifs (ITAMs) has been widely used as a fixed module to deliver a major activation signal in CAR-T construction [31]. Recently, Feucht et al. demonstrated that both the CD3ζ ITAM number and position have a notable effect on CAR-T function. They constructed different single ITAM-containing CAR-T mutants termed 1XX, X2X, and XX3 and confirmed the balanced memory and effector attributes of 1XX CAR-T cells. Corresponding clinical trials using 1XX CAR-T cells are currently underway [32]. Moreover, CD3ε, δ, and γ chains may also be effective choices for CAR design. Wu et al. first confirmed that incorporating CD3ε into CAR structures can enhance the antitumor activity of CAR-T cells. Although the mono-phosphorylated ITAM of CD3ε can reduce the cytokine production of CAR-T by recruiting inhibitory C-terminal Src kinase (Csk) kinase, it is the basic residue rich sequence (BRS) of CD3ε that enhances CAR-T persistence via p85 (a regulatory subunit of phosphoinositide 3-kinase) recruitment [33].

##### Improving Intracellular Co-Stimulatory Domain

Another important intracellular determinant of CAR-T efficacy is the costimulatory domain. Simple modification of a single co-stimulatory domain was found to be capable of increasing CAR-T persistence and function. Alteration of a single amino acid residue in CD28-based mesothelin CAR-T (changing asparagine to phenylalanine) has been reported to promote the durable antitumor control of CAR-T in pancreatic cancer xenograft models, with reduced T cell differentiation and exhaustion [34]. Another example is a recyclable 4-1BB (also called tumor necrosis factor receptor superfamily 9, TNFRSF9)-based CAR-T designed by Li et al. They first confirmed that blocking CAR ubiquitination by mutating lysine to arginine in the intracellular domain could redirect internalized CARs back to the T cell surface, leading to increased persistence and antitumor efficacy of CAR-T therapy [35]. However, different co-stimulatory domain combinations may be a favorable strategy. CD28-encoding CAR synapse recruits lymphocyte cell-specific protein tyrosine kinase (LCK) to induce antigen-independent CAR-CD3ζ phosphorylation and enhance antigen-dependent CAR-T activation, whereas the 4-1BB-encoding CAR synapse recruits the thymocyte-expressed molecule involved in selection (THEMIS)-Src-homology domain containing phosphatase-1 (SHP1) phosphatase complex to reduce CAR-CD3ζ phosphorylation. Based on these facts, CAR synapses engineered to recruit either LCK to enhance the antitumor kinetics of 4-1BB CAR-T or SHP1 to downregulate the cytokine release of CD28 CAR-T were confirmed to be helpful for enhancing CAR-T function [36]. Similarly, combining an inducible co-stimulator (ICOS) [37] or its ligand (ICOSL) [38] with 4-1BB was also found to be capable of enhancing CAR-T efficacy.

#### 2.1.3. Optimization of T Cell Selection

In view of the heterogeneity of CAR-T cells, the selection of T cell phenotypes and subtypes is also a key factor in ensuring CAR-T function. Recently, single-cell RNA sequencing (scRNA-seq) has been used to obtain the transcriptional profile of CAR-T cells [39]. Deng et al. determined the transcriptomic features of autologous CD19 CAR-T products in 24 patients with large B cell lymphoma (LBCL) by scRNA-seq and found that patients achieving complete response (CR) had three-fold higher frequencies of memory-signature-expressing CD8 T cells than patients with partial response (PR) or progressive disease (PD) [40]. NOTCH and its downstream target Forkhead box M1 (FOXM1) have been reported to be responsible for the conversion of normal CAR-T cells to stem cell memory-like CAR-T cells (CAR-iT) with superior antitumor efficacy [41].

Regarding T cell subtypes, natural killer T (NKT) cells and gamma delta T (γδT) cells engineered with CARs have exhibited significant antitumor potential. Vα24 invariant natural killer T (iNKT) cells are a unique subset of T cells expressing a canonical invariant TCR-α chain (Vα 24-Jα 18) and TCR-β chains with limited Vβ segments (Vβ11), which recognize CD1d-presented lipid antigens and induce innate and adaptive immune responses [42]. Heczey et al. constructed a CAR-iNKT called CAR.GD2 NKT targeting the GD2 ganglioside, which is an antigen highly expressed on neuroblastoma cells, and confirmed its potent antitumor activity in metastatic neuroblastoma mouse models. Unlike T cells, these CAR.GD2 NKT cells did not trigger graft-versus-host disease (GvHD) [43]. γδT is another T cell subset equipped with both innate and adaptive immune properties [44], which can function as professional antigen-presenting cells (APCs) or lyse tumor cells in an antigen-dependent/independent manner to enhance tumor control and avoid on-target off-tumor toxicity. For example, a CAR Vδ2 T has been found to be capable of presenting the processed peptide to responder alpha beta T (αβT) cells to boost tumor control [45], and an anti-GD2 CAR Vγ9Vδ2 T has been confirmed to be avoidable for the nonspecific killing of non-tumorous cells equivalently expressing GD2 but not engaging Vγ9Vδ2 TCR [46]. Moreover, CAR γδT may also be helpful in solving the antigen-loss problem as Rozenbaum et al. found that unlike general CD19 CAR-T cells, CD19 CAR γδT can also target CD19-negative leukemia cells to achieve complete tumor control, especially after zoledronate priming [47].

### 2.2. Combined Treatment Strategies of CAR-T

In response to the high tumor recurrence rate after CAR-T treatment and the limited efficacy of CAR-T in solid tumors, combined therapy has been developed to compensate for the inherent shortcomings of CAR-T cells and assist CAR-T to exert better antitumor effect. Many promising results have been reported (Table 1).

#### 2.2.1. Combination Strategies to Enhance Antigen Recognition

##### Combining CAR-T with Epigenetic Modulators

To address tumor escape caused by downregulation or loss of tumor antigen expression, the regulatory role of conventional epigenetic modulators on antigen density and distribution has been extensively studied in CAR-T treatment. Anurathapan et al. first confirmed the antigenic sensitization of epigenetic modulators in CAR-T therapy. They found that decitabine application (a hypomethylating agent that can increase tumor antigen expression by demethylating DNA) could upregulate mucin 1 (MUC1) expression in resistant CAPAN1 pancreatic cancer cells, with lower antigen expression induced by previous MUC1 CAR-T treatment, making them vulnerable to CAR-T therapy [12]. Driouk et al. demonstrated that valproic acid, an effective histone deacetylase (HDAC) inhibitor, was capable of upregulating NKG2DL expression in acute myeloid leukemia (AML) cells, thus enhancing the anti-tumor efficacy of NKG2D CAR-T cells [48]. It has been also reported that the cleavage of B cell maturation antigen (BCMA) on the surface of multiple myeloma (MM) cells and the subsequent release of soluble BCMA (sBCMA) caused by the multi-subunit γ-secretase complex (GS) is an important mechanism for reducing CAR-T efficacy. As a result, small-molecule GS inhibitors (GSIs) were found to increase BCMA and decrease sBCMA levels simultaneously and augment CAR-T efficacy in MM pre-clinical models [49]. A clinical trial investigating the feasibility and safety of applying GSIs to BCMA CAR-T therapy is ongoing (NCT03502577).

##### Combining CAR-T with Bispecific Antibodies

Bispecific T cell engager (BiTE) is a class of dual-specific antibodies consisting of two scFvs, one recruiting both CAR-T and bystander T cells by recognizing T cell surface protein CD3ε, and the other targeting a second antigen on the tumor cell surface. The feature of BiTE allows it to physically bridge tumors and T cells, thus mediating the killing of T cells to different tumor cells [65]. EGFRvIII-specific CAR-T cells were unable to eliminate heterogeneous glioblastoma but led to the proliferation of EGFRvIII^-^/EGFR^+^ glioblastoma cells [66]. In view of this, Choi et al. developed a CART.BiTE (an EGFRvIII specific CAR-T secreting EGFR-specific BiTE) and confirmed its ability to eradicate heterogeneous tumor cells in glioblastoma mouse models [50]. Combining universal CAR-T products with sequential or simultaneous delivery of bispecific adapters is another strategy. Each bispecific adapter is composed of tag (e.g., fluorescein, isothiocyanate, and biotin)-conjugated antibody fragments against individual antigens, which can be bound by corresponding anti-tag CAR-T cells. Based on this, Lohmueller et al. designed an anti-biotin CAR-T and confirmed its activation induced by biotinylated bispecific antibody (anti-CD19 and CD20)-coated tumor cells, which further mediated tumor cell cleavage and interferon (IFN)-γ production in an antibody dose-dependent manner [51]. Similarly, a cocktail of fluorescein-linked bispecific adapters has also been reported to be capable of bridging anti-fluorescent CAR-T cells with tumor cells and mediating effective eradication of heterogeneous solid tumors [52].

#### 2.2.2. Combination Strategies to Overcome the Suppressive TME

##### Combining CAR-T with Chemotherapy

The chief aim of combining CAR-T with chemotherapy is to achieve better tumor infiltration and regression by reshaping the tumor immune microenvironment. Combining CAR-T with interleukin (IL)-12 plus doxorubicin, for example, has been found to increase the deep penetration of infused T cells and boost CAR-T function in xenograft solid tumor models. Given the hypothesis that chemotherapeutic agents can stimulate tumor cells to produce T cell-attracting chemokines, Hu et al. tested different agents and found that doxorubicin could promote CXCL9/CXCL10 (produced by tumor cells)-mediated T cell penetration in the TME and that IL-12 induced high interferon (IFN)-γ expression synergistically induced chemokine production [53]. From a clinical perspective, pre-application of chemotherapeutic drugs before CAR-T infusion is also a good choice. For example, in an expanded and parallel clinical trial of anti-EGFR CAR-T (NCT01869166), pre-medication with nab-paclitaxel and cyclophosphamide showed excellent performance in enhancing CAR-T efficacy. This combination was confirmed to be the optimal stroma-depleting regimen, which could deplete tumor stroma by binding the secreted protein acidic and rich in cysteine (SPARC), thus promoting CAR-T infiltration [54].

##### Combining CAR-T with Local Therapy

Local therapy is commonly used as a typical approach to reshape the TME. Combining different local therapies with CAR-T cells has also shown great feasibility. For instance, the subsequent application of focal radiotherapy after GD2 CAR-T intravenous administration has been reported to be necessary for acquiring a complete therapeutic response in advanced syngeneic orthotopic GBM models. Intravital microscopy imaging has further indicated that radiotherapy is an important promoter of efficient vasculature-extravasation of CAR-T cells and their expansion in the TME, thus leading to a more robust and persistent anti-tumor response [55]. In view of the TME-remodeling effect of mild hyperthermia (e.g., reducing the compact stromal structure and interstitial fluid pressure and increasing blood perfusion and immune cell recruitment), concurrent CAR-T and photothermal therapy application has also shown superior CAR-T accumulation and tumor-control efficacy in solid tumor models [56].

##### Combining CAR-T with Checkpoint Blockade

As mentioned earlier, highly expressed immune and metabolic checkpoints constitute the major components of the suppressive TME. Different checkpoint blockades have been used to maintain CAR-T function in cancer treatment. With regard to the immune checkpoint, the combination of CAR-T and an immune checkpoint blockade has exhibited significant antitumor effects in both pre-clinical (e.g., combining with PD-1 and PD-L1) and clinical (e.g., combining with pembrolizumab and durvalumab) aspects [57]. Regarding the abnormal metabolic microenvironment, corresponding blockades also have the potential to maximize CAR-T efficacy [58]. Adenosine is an immunosuppressive metabolite produced at high levels in the TME, which can inhibit the immune response by binding to the adenosine 2a receptor (A2aR) expressed on immune cells. Combining CAR-T with the A2aR-specific small molecule antagonist SCH-58261 has been reported to be helpful in overcoming TME-mediated resistance and enhancing CAR-T function [67]. Similarly, Yazdanifar et al. demonstrated that combining CAR-T with inhibitors of resistant factors such as indoleamine 2,3-dioxygenases-1 (IDO1), cyclooxygenase 1/2 (COX1/2), and galectin-9 (Gal-9) also significantly augmented CAR-T efficacy in the treatment of refractory pancreatic ductal adenocarcinoma [68].

#### 2.2.3. Combining CAR-T with Vaccines to Promote In Vivo Expansion

Tumor vaccines, including whole-cell and molecular vaccines, are also important boosters for CAR-T efficacy. Whole-cell vaccines can be divided into tumor cells or dendritic cell (DC) sources. One example is a K562-derived whole-cell vaccine expressing cytomegalovirus (CMV)-pp65 protein, the immune stimulatory molecules CD40L and OX40L (CD252), and inducible safety switch caspase 9 (iC9). Caruana et al. confirmed this vaccine’s ability to augment the antitumor efficacy of CAR-redirected CMV-specific cytotoxic T cells (CTL) in xenograft tumor models [59]. For DC vaccines, Wu et al. designed an Eps8-DC vaccine by pulsing DCs with EGFR pathway substrate 8-derived peptides and found that Eps8-DCs could effectively facilitate the in vitro expansion of CAR-T cells, with an increased central memory T cell proportion and a decreased effector memory T cell proportion, and boost CAR-T function in relapsed leukemia models [60]. In addition, molecular vaccines play a significant role in promoting CAR-T expansion. A major challenge in molecular vaccine application is effective delivery to secondary lymphoid organs. Considering the “albumin hitchhiking” phenomenon (the lipid tail at one end of the molecule can bind to serum albumin, which allows the molecule to enter lymph nodes following albumin) when compounds were infused, Irvine et al. synthetized an amph-vaccine (AMP) that consists of a cargo (antigen or adjuvant) linked to a lipophilic albumin-binding tail, which could enter lymph nodes smoothly and induce a 30-fold increase in T cell priming, with better tumor control effect and lower systemic toxicity [69]. Subsequently, an AMP-CD19 vaccine was developed. It was found to be capable of inducing to a 200-fold increase in the number of infused CAR-T cells and enhancing antitumor efficacy in multiple solid tumor mouse models [61].

#### 2.2.4. Combining CAR-T with Oncolytic Viruses to Deal with Solid-Tumor Challenges

Oncolytic viruses (OVs) have been considered promising partners for cancer immunotherapy because they can affect many critical steps in the cancer-immunity cycle. OVs can enhance the antitumor immune response by directly lysing tumor cells, leading to the release of immune-activating components such as soluble antigens and oncoproteins. In addition, these OVs can also be modified to express different therapeutic genes to further increase the accumulation and function of both innate and adaptive immune cells [70]. Despite the multiple challenges encountered by CAR-T therapy in solid tumors, oncolytic viruses, especially modified ones, have exhibited remarkable synergetic and enhancing effects [71].

First, antigen delivery may fundamentally solve the problem of antigen heterogeneity. A typical example is an oncolytic vaccinia virus coding for the truncated CD19t protein (OV19t). Tumor cells infected with these OVs produce de novo CD19 on the surface, which enables the specific and efficient targeting of CD19 CAR-T cells. In turn, CAR-T-mediated tumor lysis causes the release of OV19t and further promotes the expression of CD19t in tumor cells [62]. Arming OVs with cytokines is another option. It is well known that many cytokines (e.g., IL-2, IL-15, and tumor necrosis factor [TNF]-α) are highly related to the activation and function of immune cells. Even within the immunosuppressive TME of pancreatic ductal adenocarcinoma (PDA), combining CAR-T with an oncolytic adenovirus (OAd)-expressing TNF-α and IL-2 (OAd-TNFα-IL2) has exhibited prominent antitumor effects, as OAd-TNFα-IL2 not only increased the tumor infiltration of both CAR-T and host T cells but also reshaped the TME with M1 polarization of macrophages and increased DC maturation [63]. Additionally, oncolytic viruses engineered with immune checkpoint blocking agents also presented significant synergistic effects with CAR-T in solid tumors [72]. For example, Tanoue et al. constructed an OAd with a helper-dependent Ad expressing a PD-L1 blocking mini-antibody and further confirmed its ability to significantly enhance HER2-specific CAR-T function in HER2 prostate cancer xenograft models [64]. As a promising partner for CAR-T therapy, the special “delivery system” provided by OVs has provided a new perspective for solving the challenges faced by CAR-T cells in solid tumors.

## 3. Enhanced Clinical Safety and Accessibility of CAR-T Therapy

### 3.1. Safety Maintenance of CAR-T Therapy

Adverse effects (AEs) are a major impediment to the effective application of CAR-T therapy. Life-threatening toxicity and consequent death or treatment abandonment of patients can be commonly seen in clinical trials [73]. Just like what Penack et al. have reviewed, the unique toxicity profile of CAR-T, including CRS, ICANS, cardiotoxicity, pulmonary toxicity, metabolic complications, secondary macrophage-activation syndrome (sHLH/MAS), and prolonged cytopenia, are also frequently occurring events in clinical-trial widely utilized CD19 CAR-T therapy [74]. Additionally, the personalized responses of cancer patients to CAR-T toxicity make it a more serious problem [75]. CRS, as the most common side effect of CAR-T, has characteristic clinical symptoms ranging from mild (e.g., flu-like fever, fatigue, and headache) to severe (e.g., life-threatening multi-organ system failure). Additionally, the positive feedback loop between tumor-antigen binding CAR-T produced inflammatory cytokines (e.g., TNF-α, IFN-γ) and subsequently activated bystander immune cell (e.g., macrophages and endothelial cells) released proinflammatory cytokines (e.g., IL-1 and IL-6) is considered as the main mechanism of CRS occurrence [76]. ICANS is the second most common side effect in CAR-T therapy along with CRS or subsequently, which has a wide range of clinical manifestations, such as language/behavioral disturbance, peripheral neuropathy, and acute cerebral edema [77]. Although the pathogenesis of ICANS has not been fully understood, blood–brain barrier (BBB) disruption after high-concentration proinflammatory cytokine induced endothelial activation and the diffusion of both CAR-T and cytokines into the central nervous system are thought to be the key contributors [78]. To solve these problems, many management strategies, such as accurate grading and intervention, have been adopted to minimize CAR-T toxicities [79]. Currently an increasing number of safety switches that can directly prevent or block CAR-T toxicities have been developed to help avoid AEs in clinical cancer patients without weakening CAR-T efficacy, and remarkable results were observed both preclinically and clinically [80].

#### 3.1.1. The Endogenous Witches

Inhibitory CAR (iCAR), comprised of an scFv targeting normal tissue-specific antigens and a powerful acute inhibitory signaling domain of immunoinhibitory receptors, is a promising endogenous approach to restrict unwanted AEs in CAR-T therapy. Fedorov et al. constructed a PD-1 and cytotoxic T-lymphocyte-associated protein 4 (CTLA-4)-based iCAR-T and confirmed its selective and reversible suppression of T cell activity, which provides a dynamic safety switch to prevent potential on-target off-tumor effects [81]. Another example is the killer cell immunoglobulin-like receptor (KIR)/PD-1-based iCAR (iKP CAR). The anti-CD19 CAR-T integrated with iKP CAR not only exhibited significant cytotoxicity to malignant B cells but also averted the damage of CD19-positive healthy B cells in vitro and in Burkitt’s lymphoma xenograft models [82].

The synthetic Notch (SynNotch) system is another typical endogenous safety switch. When the SynNotch part is activated by one antigen on tumor cells, the transcription of a CAR structure that recognizes a second tumor antigen is initiated [83], which allows for a specific activation of these dual-receptor AND-gate CAR-T cells by dual-antigen tumor cells [84]. In response to fatal bone marrow (containing ROR1^+^ stromal cells) failure caused by ROR1 specific CAR-T in mouse models, Srivastava et al. designed a CAR-T with a EpCAM or B7-H3 (antigens expressed on ROR1^+^ tumor cells but not ROR1^+^ stromal cells) specific synNotch receptor to enhance selectivity, which permitted both the safe targeting of tumor cells sufficiently separated from normal cells and the prevention of potential on-target off-tumor effects [85].

#### 3.1.2. The Exogenous Switches

Small-molecule drugs are the most common choice for exogenous safety switches because they can regulate cellular function by modulating protein–protein interactions. Safety switches can be divided into on and off types according to their functions, both of which have been applied to ensure CAR-T safety. Regarding the on-switches, lipocalin-based on-switch has been reported to be a suitable option for CAR-T as the human retinol binding protein 4 (hRBP4, a member of the lipocalin family) interacts with engineered hRBP4 binders in the presence of the small molecule A1120 and, thus, successfully regulated the activity of CAR-T cells [86]. Regarding the off-switches, Giordano-Attianese et al. developed a STOP-CAR-T by incorporating a chemically disruptable heterodimer (CDH) into a synthetic heterodimeric CAR. CDH has a high-affinity protein interface and can be disrupted by small-molecule drugs, which allows for the dynamic inactivation of CAR-T under timed administration [87]. Another example is an Fms-related tyrosine kinase 3 (FLT3)-specific CAR-T engineered with a rituximab-responsive off-switch, which effectively guaranteed the recovery of bone marrow after AML remission [88].

A suicide gene is also a popular choice for safety switches. The inducible caspase 9 (iCasp9) is a well-known suicide gene that has been incorporated into CAR-T cells to eliminate inappropriately activated CAR-T cells. Gargett et al. demonstrated the application of AP1903 (a small-molecule dimerizer drug) in triggering rapid apoptosis of CAR-T cells that are highly expressed by iCasp9 [89]. Another suicide gene is the herpes simplex virus type 1 thymidine kinase (HSV1-tk) gene. It has been found to be capable of inducing the suicide of transduced CAR-T cells under the administration of the prodrug ganciclovir (GCV). Incorporating CAR-T with a mutated-version HSV1-tk gene (sr39tk), Murty et al. constructed a B7H3-specific sr39tk CAR-T and confirmed its complete ablation in osteosarcoma models after the intraperitoneal administration of GCV by bioluminescence and positron emission tomography (PET) imaging [90].

In addition to these irreversible all-or-none switches, an increasing number of adjustable switches have also been devised to acquire a more flexible and accurate modulation of CAR-T activity. Through the fusion of CAR into the ligand-induced degradation (LID) domain, Richman et al. created a novel CAR-LID structure and confirmed its ability to downregulate CAR expression on demand in the presence of small-molecule ligands in CAR-T-treated tumor models. This is due to the small-molecule ligand-induced exposure of a cryptic degron within LID, which can further lead to the degradation of CAR-LID protein and the loss of CAR expression on T cells [91]. Peptide-specific switchable CAR-T (sCAR-T) is another promising choice. The bifunctional switch, consisting of a tumor antigen-specific Fab and a peptide neo-epitope, can exclusively bind to sCAR-T and regulate its activity in a dose-dependent manner. The dynamic activity modulation of CD19-specific sCAR-T in B cell leukemia xenograft models is a successful example [92]. The dose-titratable administration of the tyrosine kinase inhibitor dasatinib can also achieve dynamic regulation of CAR-T function. It can induce a function-off state in the CAR structure by restraining the phosphorylation of CD3ζ and the ζ-chain of T cell receptor-associated protein kinase 70 kDa (ZAP70), with complete recovery after discontinuation. A protective effect was confirmed in a CAR-T-treated CRS mouse model [93]. Based on the continuously updated design of safety switches, many platforms have been established to make CAR-T therapy more reliable, such as the avidity-controlled CAR platform (integrating inducible and controllable functions into CAR structures) [94], the humanized artificial receptor platform (steering CAR-T by bispecific targeting molecules) [95], and different universal CAR platforms [96,97,98].

### 3.2. Accessibility Increase of CAR-T Therapy

#### 3.2.1. Acquisition of T Cells

Currently, autologous T cells are the main source of CAR-T cells. However, the high cost and complicated acquisition process and the limited availability of T cells from immunosuppressive or immunodeficient cancer patients restrict the wide clinical application of autologous CAR-T therapy [99]. Developing techniques with both high efficiency and low cost to separate T cells is one of the solutions. DNA aptamers, for example, have been reported to be effective tools to achieve high-purity T cell isolation. Kacherovsky et al. have confirmed that DNA aptamers can help isolate pure and traceless CD8-T cells at low cost and with high yield; CAR-T cells derived from these cells were comparable to antibody-isolated CAR-T cells in terms of proliferation, phenotype, and efficacy in B cell lymphoma mouse models [100]. Exploration of allogeneic universal CAR-T cells is another potential strategy [101]. Peripheral blood mononuclear cells (PBMCs) from healthy donors, umbilical cord blood (UCB), and induced pluripotent stem cells (iPSCs) are currently the main allogeneic sources [102]. Among these, FT819, an iPSC-derived off-the-shelf CAR-T product, has emerged as a promising option [103]. It is a novel CD19 CAR-T designed by Fate Therapeutics, which encodes CAR with a single immunoreceptor tyrosine-based activation motif (1XX) to balance the effector and memory programs of CAR-T [32]. It was inserted into the T cell receptor alpha constant (TRAC) locus to delay the differentiation and exhaustion of CAR-T [104] and edited for TCR-expression elimination through the bi-allelic disruption of TRAC to mitigate the risk of GvHD [105]. According to a report at the 2019 ASH annual meeting, FT819 CAR-T has exhibited superior antitumor efficacy and survival compared to primary CD19 CAR-T in pre-clinical models, providing support for the subsequent first-of-kind phase I clinical trial [106].

#### 3.2.2. Optimization of CAR-T Manufacturing Process

Currently, viral vector-mediated semi-random DNA integration is the main approach for expressing CAR structures on T cells [107]. However, the complex and expensive manufacturing processes of viral vectors, cumbersome production, quality control steps of viral vector-transfected CAR-T, and inevitable long-term monitoring of unanticipated side effects also limit its wide clinical application. In view of this, non-viral delivery systems with low-cost and high-safety properties have emerged as a better choice [108].

PiggyBac (PB) and Sleeping Beauty (SB) transposon systems, CRISPR system (clustered regularly interspaced short palindromic repeat)-mediated site-directed integration, and mRNA carriers are all popular non-viral methods with confirmed effectiveness in CAR-T transfection; the latest improvement in each has further optimized their efficacy in CAR-T manufacturing. For PB, Bishop et al. reported that dbDNA, a minimal DNA vector lacking undesirable plasmid features, may be a viable alternative in PB-mediated CAR-T generation with lower risks and costs for clinical application [109]. Meanwhile, Querques et al. constructed a high-solubility SB transposase (hsSB) based on the crystal structure of the hyperactive SB100X variant to overcome uncontrolled transposase activity and further confirmed that hsSB could generate CAR-T cells with superior antitumor efficacy in the absence of transfection reagents [110]. CRISPR-Cas9-mediated CAR-T construction with endogenous gene disruption is another optimization. For example, a CD19 CAR-T integrating CAR to the PD1 locus not only exhibited significant antitumor efficacy in pre-clinical models but also presented safety and effectiveness in r/r aggressive B-cell non-Hodgkin lymphoma (B-NHL) patients in investigator-initiated clinical trials (IITs) [111]. Moreover, compared to the Cas9-based approach, the AAV-Cpf1 system (combining adeno-associated virus with CRISPR-Cpf1) has been reported to be more efficient in generating double knockings in CAR-T cells. An example is CD22-specific AAV-Cpf1 KIKO CAR-T, a CAR-T product with homology-directed repair knockin and immune checkpoint knockout, which has exhibited lower-level exhaustion markers but comparable tumor control effect with regard to Cas9 CAR-T [112]. Targeted mRNA nanocarriers are absorbent owing to their simplicity. By simply mixing them with lymphocytes, these mRNA nanocarriers can efficiently mediate the genome editing of CAR-T cells, during which the disruption of TCR or the transformation of the central memory phenotype can also be achieved [113].

## 4. Conclusions and Perspective

CAR-T therapy, as a promising option in tumor immunotherapy, has exhibited remarkable potential and prospects for clinical application. However, there are still some unresolved problems impeding the application of CAR-T. To enhance CAR-T efficacy in solid tumors and prevent tumor recurrence after CAR-T treatment, different CAR-T structures and combination strategies have been developed and proven to be beneficial for increasing CAR-T antitumor activity and persistence. With the continuous confirmation of the efficacy and safety in clinical transformation, these novel CAR-T therapies are expected to defeat solid tumors. To further improve the safety and accessibility of CAR-T therapy in clinics, different safeguarding and streamlined manufacturing processes have been designed and considered to provide a new perspective for reducing CAR-T toxicity and treatment expenses. From CAR-T modification and production to CAR-T combination, the updated breakthroughs are undoubtedly dispelling the clouds blocking CAR-T and blazing the light of hope. If these breakthroughs can be supported by subsequent large clinical studies, CAR-T may bring a new blueprint for cancer treatment. It is worth believing that with the deepening and broadening of CAR-T exploration, this therapy will eventually benefit clinical cancer patients to a large extent.

## Figures and Tables

**Table 1:** Combined strategies to enhance CAR-T function. CAR—Chimeric antigen receptor; MUC1—Mucin 1; NKG2D—Natural killer group 2 member D; AML—Acute myeloid leukemia; BCMA—B cell maturation antigen; sBCMA—Soluble B cell maturation antigen; MM—Multiple myeloma; BiTE—Bispecific T cell engager; EGFR—Epidermal growth factor receptor; IFN-γ—Interferon-γ; IL—Interleukin; DC—Dendritic cell; CD—Cluster of differentiation; OV—Oncolytic virus; OV19t—Truncated CD19t protein; TNF-α—Tumor necrosis factor-α; PDA—Pancreatic ductal adenocarcinoma; TME—Tumor microenvironment; OAd—Oncolytic adenovirus; HER2—Human epidermal growth factor receptor 2; PD-L1—Programmed death-ligand 1.

Combined Strategy	Impact	Tumor Model	Refs.
**Combined Strategies to Enhance Antigen Recognition**			
**Combining CAR-T with Epigenetic Modulators**			
Decitabine & MUC1-CAR-T	Upregulate MUC1 expression	Pancreatic cancer	[12]
Valproic acid & NKG2D-CAR-T	Upregulate NKG2D-ligand expression	AML	[48]
GSIs & BCMA-CAR-T	Increase BCMA and decrease sBCMA	MM	[49]
**Combining CAR-T with Bispecific Antibodies**			
EGFR-BiTE & EGFRvIII-CAR-T	Eradicate heterogeneous tumor cells	Glioblastoma	[50]
Biotinylated bispecific antibody & CAR-T	Mediate tumor lysis and IFN-γ production	Different tumors	[51]
Cocktail of fluorescein-bispecific adapters & CAR-T	Eradicate heterogeneous tumor cells	Solid tumors	[52]
**Combined Strategies to Overcome the Suppressive TME**			
**Combining CAR-T with Chemotherapy**			
IL-12 plus doxorubicin & CAR-T	Increase T-cell deep penetration	Solid tumors	[53]
Nab-paclitaxel plus cyclophosphamide & CAR-T	Promote CAR-T infiltration	Biliary tract cancer	[54]
**Combining CAR-T with local therapy**			
Focal radiotherapy & GD2 CAR-T	Promote CAR-T extravasation and expansion	Glioblastoma	[55]
Photothermal therapy & CAR-T	Promote CAR-T accumulation	Solid tumors	[56]
**Combining CAR-T with checkpoint blockade**			
Immune checkpoint blockade & CAR-T	Maximize CAR-T efficacy	Solid tumors	[57]
Metabolic checkpoint blockade & CAR-T	Maximize CAR-T efficacy	Solid tumors	[58]
**Combined strategies to promote in vivo expansion**			
Whole-cell vaccine (tumor/DC) & CAR-T	Boost CAR-T function	Different tumors	[59,60]
Molecular vaccine & CAR-T	Increase CAR-T expansion	Solid tumors	[61]
**Combined strategies to deal with solid-tumor challenges**			
OV19t & CD19-CAR-T	Deliver antigen and overcome heterogeneity	Solid tumors	[62]
OAd-TNFα-IL2 & CAR-T	Increase T-cell infiltration and reshape TME	PDA	[63]
OAd-expressing PD-L1 mini-body & HER2-CAR-T	Enhance CAR-T function	Prostate cancer	[64]

## References

[1] Sheridan C. (2017). First approval in sight for Novartis’ CAR-T therapy after panel vote. Nat. Biotechnol..

[2] Locke F.L., Go W.Y., Neelapu S.S. (2020). Development and Use of the Anti-CD19 Chimeric Antigen Receptor T-Cell Therapy Axicabtagene Ciloleucel in Large B-Cell Lymphoma A Review. JAMA Oncol..

[3] Slaney C.Y., Kershaw M.H., Darcy P.K. (2014). Trafficking of T Cells into Tumors. Cancer Res..

[4] Baluk P., Hashizume H., McDonald D.M. (2005). Cellular abnormalities of blood vessels as targets in cancer. Curr. Opin. Genet. Dev..

[5] Henke E., Nandigama R., Ergün S. (2020). Extracellular Matrix in the Tumor Microenvironment and Its Impact on Cancer Therapy. Front. Mol. Biosci..

[6] Burga R.A., Thorn M., Point G.R., Guha P., Nguyen C.T., Licata L.A., DeMatteo R.P., Ayala A., Espat N.J., Junghans R.P. (2015). Liver myeloid-derived suppressor cells expand in response to liver metastases in mice and inhibit the anti-tumor efficacy of anti-CEA CAR-T. Cancer Immunol. Immunother..

[7] Budhu S., Schaer D.A., Li Y., Toledo-Crow R., Panageas K., Yang X., Zhong H., Houghton A.N., Silverstein S.C., Merghoub T. (2017). Blockade of surface-bound TGF-beta on regulatory T cells abrogates suppression of effector T cell function in the tumor microenvironment. Sci. Signal..

[8] Hu W., Zi Z., Jin Y., Li G., Shao K., Cai Q., Ma X., Wei F. (2019). CRISPR/Cas9-mediated PD-1 disruption enhances human mesothelin-targeted CAR T cell effector functions. Cancer Immunol. Immunother..

[9] Berahovich R., Liu X., Zhou H., Tsadik E., Xu S., Golubovskaya V., Wu L. (2019). Hypoxia Selectively Impairs CAR-T Cells in vitro. Cancers.

[10] Cascone T., McKenzie J.A., Mbofung R.M., Punt S., Wang Z., Xu C., Williams L.J., Wang Z., Bristow C.A., Carugo A. (2018). Increased Tumor Glycolysis Characterizes Immune Resistance to Adoptive T Cell Therapy. Cell Metab..

[11] Chen N., Li X., Chintala N.K., Tano Z.E., Adusumilli P.S. (2018). Driving CARs on the uneven road of antigen heterogeneity in solid tumors. Curr. Opin. Immunol..

[12] Anurathapan U., Chan R.C., Hindi H.F., Mucharla R., Bajgain P., Hayes B.C., Fisher W.E., Heslop H.E., Rooney C.M., Brenner M.K. (2014). Kinetics of Tumor Destruction by Chimeric Antigen Receptor-modified T Cells. Mol. Ther..

[13] Lesch S., Benmebarek M.-R., Cadilha B.L., Stoiber S., Subklewe M., Endres S., Kobold S. (2020). Determinants of response and resistance to CAR T cell therapy. Semin. Cancer Biol..

[14] Chou C.K., Turtle C.J. (2019). Insight into mechanisms associated with cytokine release syndrome and neurotoxicity after CD19 CAR-T cell immunotherapy. Bone Marrow Transplant..

[15] Morgan R.A., Yang J.C., Kitano M., Dudley M.E., Laurencot C.M., Rosenberg S.A. (2010). Case Report of a Serious Adverse Event Following the Administration of T Cells Transduced With a Chimeric Antigen Receptor Recognizing ERBB2. Mol. Ther..

[16] Poorebrahim M., Sadeghi S., Fakhr E., Abazari M.F., Poortahmasebi V., Kheirollahi A., Askari H., Rajabzadeh A., Rastegarpanah M., Linē A. (2019). Production of CAR T-cells by GMP-grade lentiviral vectors: Latest advances and future prospects. Crit. Rev. Clin. Lab. Sci..

[17] Gee A.P. (2018). GMP CAR-T cell production. Best Pract. Res. Clin. Haematol..

[18] Dai X., Mei Y., Cai D., Han W. (2019). Standardizing CAR-T therapy: Getting it scaled up. Biotechnol. Adv..

[19] Magee J.A., Piskounova E., Morrison S.J. (2012). Cancer Stem Cells: Impact, Heterogeneity, and Uncertainty. Cancer Cell.

[20] Vora P., Venugopal C., Salim S.K., Tatari N., Bakhshinyan D., Singh M., Seyfrid M., Upreti D., Rentas S., Wong N. (2020). The Rational Development of CD133-Targeting Immunotherapies for Glioblastoma. Cell Stem Cell.

[21] Yang D., Sun B., Dai H., Li W., Shi L., Zhang P., Li S., Zhao X. (2019). T cells expressing NKG2D chimeric antigen receptors efficiently eliminate glioblastoma and cancer stem cells. J. Immunother. Cancer.

[22] Wang L., Yin E.T.S., Zhao H., Ni F., Hu Y., Huang H. (2020). CAR-T cells: The Chinese experience. Expert Opin. Biol. Ther..

[23] Wang N., Hu X.L., Cao W.Y., Li C.R., Xiao Y., Cao Y., Gu C.J., Zhang S.K., Chen L.T., Cheng J.L. (2020). Efficacy and safety of CAR19/22 T-cell cocktail therapy in patients with refractory/relapsed B-cell malignancies. Blood.

[24] Pan J., Zuo S., Deng B., Xu X., Li C., Zheng Q., Ling Z., Song W., Xu J., Duan J. (2020). Sequential CD19-22 CAR T therapy induces sustained remission in children with r/r B-ALL. Blood.

[25] Dannenfelser R., Allen G.M., VanderSluis B., Koegel A.K., Levinson S., Stark S.R., Yao V., Tadych A., Troyanskaya O.G., Lim W.A. (2020). Discriminatory Power of Combinatorial Antigen Recognition in Cancer T Cell Therapies. Cell Syst..

[26] Ruella M., Barrett D.M., Kenderian S.S., Shestova O., Hofmann T.J., Perazzelli J., Klichinsky M., Aikawa V., Nazimuddin F., Kozlowski M. (2016). Dual CD19 and CD123 targeting prevents antigen-loss relapses after CD19-directed immunotherapies. J. Clin. Investig..

[27] Ahn S., Li J., Sun C., Gao K., Hirabayashi K., Li H., Savoldo B., Liu R., Dotti G. (2019). Cancer Immunotherapy with T Cells Carrying Bispecific Receptors That Mimic Antibodies. Cancer Immunol. Res..

[28] Fousek K., Watanabe J., Joseph S.K., George A., An X., Byrd T.T., Morris J.S., Luong A., Martínez-Paniagua M.A., Sanber K. (2021). CAR T-cells that target acute B-lineage leukemia irrespective of CD19 expression. Leukemia.

[29] Huang R., Li X., He Y., Zhu W., Gao L., Liu Y., Wen Q., Zhong J.F., Zhang C., Zhang X. (2020). Recent advances in CAR-T cell engineering. J. Hematol. Oncol..

[30] Dong D., Zheng L., Lin J., Zhang B., Zhu Y., Li N., Xie S., Wang Y., Gao N., Huang Z. (2019). Structural basis of assembly of the human T cell receptor–CD3 complex. Nat. Cell Biol..

[31] Lindner S.E., Johnson S.M., Brown C.E., Wang L.D. (2020). Chimeric antigen receptor signaling: Functional consequences and design implications. Sci. Adv..

[32] Feucht J., Sun J., Eyquem J., Ho Y.-J., Zhao Z., Leibold J., Dobrin A., Cabriolu A., Hamieh M., Sadelain M. (2019). Calibration of CAR activation potential directs alternative T cell fates and therapeutic potency. Nat. Med..

[33] Wu W., Zhou Q., Masubuchi T., Shi X., Li H., Xu X., Huang M., Meng L., He X., Zhu H. (2020). Multiple Signaling Roles of CD3epsilon and Its Application in CAR-T Cell Therapy. Cell.

[34] Hsieh E.M., Scherer L.D., Rouce R.H. (2020). Replacing CAR-T cell resistance with persistence by changing a single residue. J. Clin. Investig..

[35] Li W., Qiu S., Chen J., Jiang S., Chen W., Jiang J., Wang F., Si W., Shu Y., Wei P. (2020). Chimeric Antigen Receptor Designed to Prevent Ubiquitination and Downregulation Showed Durable Antitumor Efficacy. Immunity.

[36] Sun C., Shou P., Du H., Hirabayashi K., Chen Y., Herring L.E., Ahn S., Xu Y., Suzuki K., Li G. (2020). THEMIS-SHP1 Recruitment by 4-1BB Tunes LCK-Mediated Priming of Chimeric Antigen Receptor-Redirected T Cells. Cancer Cell.

[37] Guedan S., Posey A.D., Shaw C., Wing A., Da T., Patel P.R., Mcgettigan S.E., Casado-Medrano V., Kawalekar O.U., Uribe-Herranz M. (2018). Enhancing CAR T cell persistence through ICOS and 4-1BB costimulation. JCI Insight.

[38] Hu W., Huang X., Huang X., Chen W., Hao L., Chen Z. (2019). Chimeric antigen receptor modified T cell (CAR-T) co-expressed with ICOSL-41BB promote CAR-T proliferation and tumor rejection. Biomed. Pharmacother..

[39] Sheih A., Voillet V., Hanafi L.-A., DeBerg H.A., Yajima M., Hawkins R., Gersuk V., Riddell S.R., Maloney D.G., Wohlfahrt M.E. (2020). Clonal kinetics and single-cell transcriptional profiling of CAR-T cells in patients undergoing CD19 CAR-T immunotherapy. Nat. Commun..

[40] Deng Q., Han G., Puebla-Osorio N., Ma M.C.J., Strati P., Chasen B., Dai E., Dang M., Jain N., Yang H. (2020). Characteristics of anti-CD19 CAR T cell infusion products associated with efficacy and toxicity in patients with large B cell lymphomas. Nat. Med..

[41] Kondo T., Ando M., Nagai N., Tomisato W., Srirat T., Liu B., Mise-Omata S., Ikeda M., Chikuma S., Nishimasu H. (2019). The NOTCH–FOXM1 Axis Plays a Key Role in Mitochondrial Biogenesis in the Induction of Human Stem Cell Memory–like CAR-T Cells. Cancer Res..

[42] Brennan P.J., Brigl M., Brenner M.B. (2013). Invariant natural killer T cells: An innate activation scheme linked to diverse effector functions. Nat. Rev. Immunol..

[43] Heczey A., Liu D., Tian G., Courtney A.N., Wei J., Marinova E., Gao X., Guo L., Yvon E., Hicks J. (2014). Invariant NKT cells with chimeric antigen receptor provide a novel platform for safe and effective cancer immunotherapy. Blood.

[44] Fisher J., Anderson J. (2018). Engineering Approaches in Human Gamma Delta T Cells for Cancer Immunotherapy. Front. Immunol..

[45] Capsomidis A., Benthall G., Van Acker H.H., Fisher J., Kramer A.M., Abeln Z., Majani Y., Gileadi T., Wallace R., Gustafsson K. (2018). Chimeric Antigen Receptor-Engineered Human Gamma Delta T Cells: Enhanced Cytotoxicity with Retention of Cross Presentation. Mol. Ther..

[46] Fisher J., Abramowski P., Don N.D.W., Flutter B., Capsomidis A., Cheung G.W.-K., Gustafsson K., Anderson J. (2017). Avoidance of On-Target Off-Tumor Activation Using a Co-stimulation-Only Chimeric Antigen Receptor. Mol. Ther..

[47] Rozenbaum M., Meir A., Aharony Y., Itzhaki O., Schachter J., Bank I., Jacoby E., Besser M.J. (2020). Gamma-Delta CAR-T Cells Show CAR-Directed and Independent Activity Against Leukemia. Front. Immunol..

[48] Driouk L., Gicobi J., Kamihara Y., Rutherford K., Dranoff G., Ritz J., Baumeister S.H.C. (2019). Chimeric Antigen Receptor T Cells Targeting NKG2D-Ligands Show Robust Efficacy Against Acute Myeloid Leukemia and T-Cell Acute Lymphoblastic Leukemia. Blood.

[49] Pont M.J., Hill T., Cole G.O., Abbott J.J., Kelliher J., Salter A.I., Hudecek M., Comstock M.L., Rajan A., Patel B.K.R. (2019). gamma-Secretase inhibition increases efficacy of BCMA-specific chimeric antigen receptor T cells in multiple myeloma. Blood.

[50] Choi B.D., Yu X., Castano A.P., Bouffard A.A., Schmidts A., Larson R.C., Bailey S.R., Boroughs A.C., Frigault M.J., Leick M.B. (2019). CAR-T cells secreting BiTEs circumvent antigen escape without detectable toxicity. Nat. Biotechnol..

[51] Lohmueller J.J., Ham J.D., Kvorjak M., Finn O.J. (2018). mSA2 affinity-enhanced biotin-binding CAR T cells for universal tumor targeting. OncoImmunology.

[52] Lee Y.G., Marks I., Srinivasarao M., Kanduluru A.K., Mahalingam S.M., Liu X., Chu H., Low P.S. (2018). Use of a Single CAR T Cell and Several Bispecific Adapters Facilitates Eradication of Multiple Antigenically Different Solid Tumors. Cancer Res..

[53] Hu J., Sun C., Bernatchez C., Xia X., Hwu P., Dotti G., Li S. (2018). T-cell Homing Therapy for Reducing Regulatory T Cells and Preserving Effector T-cell Function in Large Solid Tumors. Clin. Cancer Res..

[54] Guo Y., Feng K.-C., Liu Y., Wu Z., Dai H., Yang Q.-M., Wang Y., Jia H., Han W. (2018). Phase I Study of Chimeric Antigen Receptor–Modified T Cells in Patients with EGFR-Positive Advanced Biliary Tract Cancers. Clin. Cancer Res..

[55] Murty S., Haile S.T., Beinat C., Aalipour A., Alam I.S., Murty T., Shaffer T.M., Patel C.B., Graves E.E., Mackall C.L. (2020). Intravital imaging reveals synergistic effect of CAR T-cells and radiation therapy in a preclinical immunocompetent glioblastoma model. OncoImmunology.

[56] Chen Q., Hu Q.Y., Dukhovlinova E., Chen G.J., Ahn S., Wang C., Ogunnaike E.A., Ligler F.S., Dotti G., Gu Z. (2019). Photothermal Therapy Promotes Tumor Infiltration and Antitumor Activity of CAR T Cells. Adv. Mater..

[57] Grosser R., Cherkassky L., Chintala N., Adusumilli P.S. (2019). Combination Immunotherapy with CAR T Cells and Checkpoint Blockade for the Treatment of Solid Tumors. Cancer Cell.

[58] Xu X., Gnanaprakasam J.N.R., Sherman J., Wang R. (2019). A Metabolism Toolbox for CAR T Therapy. Front. Oncol..

[59] Caruana I., Weber G., Ballard B.C., Wood M.S., Savoldo B., Dotti G. (2015). K562-Derived Whole-Cell Vaccine Enhances Antitumor Responses of CAR-Redirected Virus-Specific Cytotoxic T Lymphocytes In Vivo. Clin. Cancer Res..

[60] Wu M., Zhang L., Zhang H., Ning J., Tu S., He Y., Li Y. (2019). CD19 chimeric antigen receptor–redirected T cells combined with epidermal growth factor receptor pathway substrate 8 peptide–derived dendritic cell vaccine in leukemia. Cytotherapy.

[61] Ma L., Dichwalkar T., Chang J.Y.H., Cossette B., Garafola D., Zhang A.Q., Fichter M., Wang C., Liang S., Silva M. (2019). Enhanced CAR-T cell activity against solid tumors by vaccine boosting through the chimeric receptor. Science.

[62] Park A.K., Fong Y., Kim S.-I., Yang J., Murad J.P., Lu J., Jeang B., Chang W.-C., Chen N.G., Thomas S.H. (2020). Effective combination immunotherapy using oncolytic viruses to deliver CAR targets to solid tumors. Sci. Transl. Med..

[63] Watanabe K., Luo Y., Da T., Guedan S., Ruella M., Scholler J., Keith B., Young R.M., Engels B., Sorsa S. (2018). Pancreatic cancer therapy with combined mesothelin-redirected chimeric antigen receptor T cells and cytokine-armed oncolytic adenoviruses. JCI Insight.

[64] Tanoue K., Shaw A.R., Watanabe N., Porter C., Rana B., Gottschalk S., Brenner M., Suzuki M. (2017). Armed Oncolytic Adenovirus–Expressing PD-L1 Mini-Body Enhances Antitumor Effects of Chimeric Antigen Receptor T Cells in Solid Tumors. Cancer Res..

[65] Goebeler M.-E., Bargou R.C. (2020). T cell-engaging therapies—BiTEs and beyond. Nat. Rev. Clin. Oncol..

[66] O’Rourke D.M., Nasrallah M.P., Desai A., Melenhorst J.J., Mansfield K., Morrissette J.J.D., Martinez-Lage M., Brem S., Maloney E., Shen A. (2017). A single dose of peripherally infused EGFRvIII-directed CAR T cells mediates antigen loss and induces adaptive resistance in patients with recurrent glioblastoma. Sci. Transl. Med..

[67] Masoumi E., Jafarzadeh L., Mirzaei H.R., Alishah K., Fallah-Mehrjardi K., Rostamian H., Khakpoor-Koosheh M., Meshkani R., Noorbakhsh F., Hadjati J. (2020). Genetic and pharmacological targeting of A2a receptor improves function of anti-mesothelin CAR T cells. J. Exp. Clin. Cancer Res..

[68] Yazdanifar M., Zhou R., Grover P., Williams C., Bose M., Moore L.J., Wu S.T., Maher J., Dreau D., Mukherjee P. (2019). Overcoming Immunological Resistance Enhances the Efficacy of a Novel Anti-tMUC1-CAR T Cell Treatment against Pancreatic Ductal Adenocarcinoma. Cells.

[69] Liu H., Moynihan K.D., Zheng Y., Szeto G.L., Li A.V., Huang B., Van Egeren D.S., Park C., Irvine D.J. (2014). Structure-based programming of lymph-node targeting in molecular vaccines. Nat. Cell Biol..

[70] Bommareddy P.K., Shettigar M., Kaufman H.L. (2018). Integrating oncolytic viruses in combination cancer immunotherapy (vol 18, pg 498, 2018). Nat. Rev. Immunol..

[71] Guedan S., Alemany R. (2018). CAR-T Cells and Oncolytic Viruses: Joining Forces to Overcome the Solid Tumor Challenge. Front. Immunol..

[72] Harrington K., Freeman D.J., Kelly B., Harper J., Soria J.-C. (2019). Optimizing oncolytic virotherapy in cancer treatment. Nat. Rev. Drug Discov..

[73] Neelapu S.S., Tummala S., Kebriaei P., Wierda W., Gutierrez C., Locke F.L., Komanduri K.V., Lin Y., Jain N., Daver N. (2018). Chimeric antigen receptor T-cell therapy—assessment and management of toxicities. Nat. Rev. Clin. Oncol..

[74] Penack O., Koenecke C. (2020). Complications after CD19+ CAR T-Cell Therapy. Cancers.

[75] Oved J.H., Barrett D.M., Teachey D.T. (2019). Cellular therapy: Immune-related complications. Immunol. Rev..

[76] Xu X.-J., Tang Y.-M. (2014). Cytokine release syndrome in cancer immunotherapy with chimeric antigen receptor engineered T cells. Cancer Lett..

[77] Gust J., Taraseviciute A., Turtle C.J. (2018). Neurotoxicity Associated with CD19-Targeted CAR-T Cell Therapies. CNS Drugs.

[78] Gust J., Ponce R., Liles W.C., Garden G.A., Turtle C.J. (2020). Cytokines in CAR T Cell–Associated Neurotoxicity. Front. Immunol..

[79] Schubert M.-L., Schmitt M., Wang L., Ramos C., Jordan K., Müller-Tidow C., Dreger P. (2021). Side-effect management of chimeric antigen receptor (CAR) T-cell therapy. Ann. Oncol..

[80] Yu S., Yi M., Qin S., Wu K. (2019). Next generation chimeric antigen receptor T cells: Safety strategies to overcome toxicity. Mol. Cancer.

[81] Fedorov V.D., Themeli M., Sadelain M. (2013). PD-1- and CTLA-4-Based Inhibitory Chimeric Antigen Receptors (iCARs) Divert Off-Target Immunotherapy Responses. Sci. Transl. Med..

[82] Tao L., Farooq M.A., Gao Y.X., Zhang L., Niu C.Y., Ajmal I., Zhou Y., He C., Zhao G.X., Yao J. (2020). CD19-CAR-T Cells Bearing a KIR/PD-1-Based Inhibitory CAR Eradicate CD19(+)HLA-C1(-) Malignant B Cells While Sparing CD19(+)HLA-C1(+) Healthy B Cells. Cancers.

[83] Morsut L., Roybal K.T., Xiong X., Gordley R.M., Coyle S.M., Thomson M., Lim W.A. (2016). Engineering Customized Cell Sensing and Response Behaviors Using Synthetic Notch Receptors. Cell.

[84] Roybal K.T., Rupp L.J., Morsut L., Walker W.J., McNally K.A., Park J.S., Lim W.A. (2016). Precision Tumor Recognition by T Cells With Combinatorial Antigen-Sensing Circuits. Cell.

[85] Srivastava S., Salter A.I., Liggitt D., Yechan-Gunja S., Sarvothama M., Cooper K., Smythe K.S., Dudakov J.A., Pierce R.H., Rader C. (2019). Logic-Gated ROR1 Chimeric Antigen Receptor Expression Rescues T Cell-Mediated Toxicity to Normal Tissues and Enables Selective Tumor Targeting. Cancer Cell.

[86] Zajc C.U., Dobersberger M., Schaffner I., Mlynek G., Puhringer D., Salzer B., Djinovic-Carugo K., Steinberger P., Linhares A.D., Yang N.J. (2020). A conformation-specific ON-switch for controlling CAR T cells with an orally available drug. Proc. Natl. Acad. Sci. USA.

[87] Giordano-Attianese G., Gainza P., Gray-Gaillard E., Cribioli E., Shui S., Kim S., Kwak M.-J., Vollers S., Osorio A.D.J.C., Reichenbach P. (2020). A computationally designed chimeric antigen receptor provides a small-molecule safety switch for T-cell therapy. Nat. Biotechnol..

[88] Sommer C., Cheng H.-Y., Nguyen D., Dettling D., Yeung Y.A., Sutton J., Hamze M., Valton J., Smith J., Djuretic I. (2020). Allogeneic FLT3 CAR T Cells with an Off-Switch Exhibit Potent Activity against AML and Can Be Depleted to Expedite Bone Marrow Recovery. Mol. Ther..

[89] Gargett T., Brown M.P. (2014). The inducible caspase-9 suicide gene system as a "safety switch" to limit on-target, off-tumor toxicities of chimeric antigen receptor T cells. Front. Pharmacol..

[90] Murty S., Labanieh L., Murty T., Gowrishankar G., Haywood T., Alam I.S., Beinat C., Robinson E., Aalipour A., Klysz D.D. (2020). PET Reporter Gene Imaging and Ganciclovir-Mediated Ablation of Chimeric Antigen Receptor T Cells in Solid Tumors. Cancer Res..

[91] Richman S.A., Wang L.-C., Moon E.K., Khire U.R., Albelda S.M., Milone M.C. (2020). Ligand-Induced Degradation of a CAR Permits Reversible Remote Control of CAR T Cell Activity In Vitro and In Vivo. Mol. Ther..

[92] Rodgers D.T., Mazagova M., Hampton E.N., Cao Y., Ramadoss N.S., Hardy I.R., Schulman A., Du J., Wang F., Singer O. (2016). Switch-mediated activation and retargeting of CAR-T cells for B-cell malignancies. Proc. Natl. Acad. Sci. USA.

[93] Mestermann K., Giavridis T., Weber J., Rydzek J., Frenz S., Nerreter T., Mades A., Sadelain M., Einsele H., Hudecek M. (2019). The tyrosine kinase inhibitor dasatinib acts as a pharmacologic on/off switch for CAR T cells. Sci. Transl. Med..

[94] Salzer B., Schueller C.M., Zajc C.U., Peters T., Schoeber M.A., Kovacic B., Buri M.C., Lobner E., Dushek O., Huppa J.B. (2020). Engineering AvidCARs for combinatorial antigen recognition and reversible control of CAR function. Nat. Commun..

[95] Feldmann A., Hoffmann A., Bergmann R., Koristka S., Berndt N., Arndt C., Loureiro L.R., Kittel-Boselli E., Mitwasi N., Kegler A. (2020). Versatile chimeric antigen receptor platform for controllable and combinatorial T cell therapy. OncoImmunology.

[96] Albert S., Arndt C., Feldmann A., Bergmann R., Bachmann D., Koristka S., Ludwig F., Ziller-Walter P., Kegler A., Gärtner S. (2017). A novel nanobody-based target module for retargeting of T lymphocytes to EGFR-expressing cancer cells via the modular UniCAR platform. OncoImmunology.

[97] Kim M.S., Ma J.S.Y., Yun H., Cao Y., Kim J.Y., Chi V., Wang D., Woods A., Sherwood L., Caballero D. (2015). Redirection of Genetically Engineered CAR-T Cells Using Bifunctional Small Molecules. J. Am. Chem. Soc..

[98] Ma J.S.Y., Kim J.Y., Kazane S.A., Choi S.-H., Yun H.Y., Kim M.S., Rodgers D.T., Pugh H.M., Singer O., Sun S.B. (2016). Versatile strategy for controlling the specificity and activity of engineered T cells. Proc. Natl. Acad. Sci. USA.

[99] Roddie C., O’Reilly M., Pinto J.D.A., Vispute K., Lowdell M. (2019). Manufacturing chimeric antigen receptor T cells: Issues and challenges. Cytotherapy.

[100] Kacherovsky N., Cardle I.I., Cheng E.L., Yu J.L., Baldwin M.L., Salipante S.J., Jensen M.C., Pun S.H. (2019). Traceless aptamer-mediated isolation of CD8+ T cells for chimeric antigen receptor T-cell therapy. Nat. Biomed. Eng..

[101] Zhao J., Lin Q., Song Y., Liu D. (2018). Universal CARs, universal T cells, and universal CAR T cells. J. Hematol. Oncol..

[102] Depil S., Duchateau P., Grupp S.A., Mufti G., Poirot L. (2020). ‘Off-the-shelf’ allogeneic CAR T cells: Development and challenges. Nat. Rev. Drug Discov..

[103] (2018). From Pluripotent Stem to CAR T Cells. Cancer Discov..

[104] Eyquem J., Mansilla-Soto J., Giavridis T., Van Der Stegen S.J.C., Hamieh M., Cunanan K.M., Odak A., Gönen K.M.C.M., Sadelain J.E.J.M.-S.T.G.S.J.C.V.D.S.M.H.A.O.M. (2017). Targeting a CAR to the TRAC locus with CRISPR/Cas9 enhances tumour rejection. Nat. Cell Biol..

[105] Stenger D., Stief T.A., Kaeuferle T., Willier S., Rataj F., Schober K., Vick B., Lotfi R., Wagner B., Grünewald T.G.P. (2020). Endogenous TCR promotes in vivo persistence of CD19-CAR-T cells compared to a CRISPR/Cas9-mediated TCR knockout CAR. Blood.

[106] Chang C., Van Der Stegen S., Mili M., Clarke R., Lai Y.-S., Witty A., Lindenbergh P., Yang B.-H., Husain M., Shaked H. (2019). FT819: Translation of Off-the-Shelf TCR-Less Trac-1XX CAR-T Cells in Support of First-of-Kind Phase I Clinical Trial. Blood.

[107] Ferreira M.V., Cabral E.T., Coroadinha A.S. (2021). Progress and Perspectives in the Development of Lentiviral Vector Producer Cells. Biotechnol. J..

[108] Harris E., Elmer J.J. (2021). Optimization of electroporation and other non-viral gene delivery strategies for T cells. Biotechnol. Prog..

[109] Bishop D.C., Caproni L., Gowrishankar K., Legiewicz M., Karbowniczek K., Tite J., Gottlieb D.J., Micklethwaite K.P. (2020). CAR T Cell Generation by piggyBac Transposition from Linear Doggybone DNA Vectors Requires Transposon DNA-Flanking Regions. Mol. Ther. Methods Clin. Dev..

[110] Querques I., Mades A., Zuliani C., Miskey C., Alb M., Grueso E., Machwirth M., Rausch T., Einsele H., Ivics Z. (2019). A highly soluble Sleeping Beauty transposase improves control of gene insertion. Nat. Biotechnol..

[111] Zhang J., Hu Y., Yang J., Li W., Tian Y., Wei G., Zhang L., Zhao K., Qi Y., Tan B. (2020). Development and clinical evaluation of non-viral genome specific targeted CAR T cells in relapsed/refractory B-cell non-Hodgkin lymphoma. medRxiv.

[112] Dai X., Park J.J., Du Y., Kim H.R., Wang G., Errami Y., Chen S. (2019). One-step generation of modular CAR-T cells with AAV–Cpf1. Nat. Methods.

[113] Moffett H.F., Coon M.E., Radtke S., Stephan S.B., McKnight L., Lambert A., Stoddard B.L., Kiem H.P., Stephan M.T. (2017). Hit-and-run programming of therapeutic cytoreagents using mRNA nanocarriers. Nat. Commun..

